# Risk factors for hypoglycemia in hospitalized patients with diabetes: a retrospective analysis of electronic medical record data

**DOI:** 10.3389/fphar.2026.1741870

**Published:** 2026-03-26

**Authors:** Liren Li, Jiayu Chen, Tongshu Guan, Ping Zheng

**Affiliations:** 1 Clinical Pharmacy Center, Nanfang Hospital, Southern Medical University, Guangzhou, China; 2 Department of Pharmacy, Nanfang Hospital, Southern Medical University, Guangzhou, China

**Keywords:** diabetes mellitus, hospitalized patients, hypoglycemia, risk factor, temporal pattern

## Abstract

**Objective:**

To evaluate the incidence of hypoglycemia events in hospitalized patients with diabetes, identify factors associated with hypoglycemia, and analyze hypoglycemia events to provide a scientific basis for the precise nursing care of inpatients with hypoglycemia.

**Method:**

We conducted a retrospective cohort study using the hospital’s medical database from 2021 to 2022. We collected data from 28,580 patients with diabetes to assess the incidence of hypoglycemia during hospitalization. We analyzed the temporal characteristics of hypoglycemia and evaluated its relationships with patient demographics and behavioral characteristics, medications, comorbidities, and laboratory parameters.

**Outcome:**

Hypoglycemia occurred in 3,477 (12.2%) of the 28,580 enrolled inpatients. Multivariable analysis identified T1DM, impaired renal function, and insulin or glinide use as factors significantly associated with hypoglycemia. Factors associated with lower odds of hypoglycemia included older age, higher body mass index, higher average glucose level, and the use of SGLT-2 or DPP-4 inhibitors. Temporal analysis revealed distinct patterns: hypoglycemia in patients with T1DM peaks within 3 days of admission, while in patients with T2DM, it also peaks within 3 days but with a lower overall incidence.

**Conclusion:**

This study underscores the clinical significance of hypoglycemia in hospitalized patients with diabetes. The elucidated risk factors and temporal characteristics of hypoglycemic events form the basis for devising proactive management strategies to reduce its occurrence.

## 1 Introduction

Diabetes is a chronic condition characterized by hyperglycemia, necessitating diligent blood glucose management to prevent long-term complications (American Diabetes Association, 2011). While controlling hyperglycemia is a cornerstone of diabetes care, the therapeutic process is often complicated by hypoglycemia, the most common adverse effect of glucose-lowering treatment (Carey et al., 2013). Severe hypoglycemic events are particularly concerning as they can lead to life-threatening complications, including arrhythmias, neurological damage, and cardiovascular events (Yun and Ko, 2015; Pathak et al., 2016). Moreover, hypoglycemia is consistently associated with increased hospitalization duration, healthcare costs, and all-cause mortality (Amiel, 2021; Lee et al., 2018).

The scale of this problem is substantial. A systematic review of 46 studies (n = 532,542) revealed that 51% of patients with type 2 diabetes mellitus (T2DM) experience hypoglycemia, averaging 20 episodes per year (Edridge et al., 2015). In China, the reported incidence ranges from 32% to 41% for T2DM and rises to 75% for patients with type 1 diabetes (T1DM) (Wu et al., 2021; Chen et al., 2023). Within the evolving paradigm of personalized glucose management, hypoglycemia has emerged as a primary obstacle to achieving optimal glycemic targets, underscoring the critical importance of its prevention (American Diabetes Association, 2020).

The risk of hypoglycemia is markedly elevated in the inpatient setting. Hospitalized patients often present with a confluence of predisposing factors, including fragile health status, psychological stress, unpredictable nutritional intake (e.g., fasting for procedures) and frequent adjustments to glucose-lowering regimens by different care teams (Witte et al., 2022; Khazai and Hamdy, 2016; Pratiwi et al., 2020). These factors contribute to significant blood glucose fluctuations and a substantially higher incidence of hypoglycemia. Data from the United Kingdom National Diabetes Inpatient Audit consistently show that approximately one-fifth of hospitalized diabetes patients experience hypoglycemia on any given day—an incidence nearly double that observed in the community-dwelling diabetes population (Holt, 2020). This high rate of in-hospital hypoglycemia not only directly increases patient morbidity and prolongs hospital stays but may also counteract the long-term benefits of glycemic control (Brutsaert et al., 2014; Cruz, 2020; Turchin et al., 2009). Consequently, identifying and mitigating the risk factors for hypoglycemia in this vulnerable population is a clinical imperative.

Previous research has identified several risk factors associated with in-hospital hypoglycemia. Key elements include having T1DM, advanced age (e.g., >75 years), the presence of multiple comorbidities, and the use of specific antidiabetic agents such as insulin and sulfonylureas(SU) (American Diabetes Association, 2020; Ligthelm et al., 2012; Kim et al., 2011; Davis et al., 2010; Kostev et al., 2014; Bruderer et al., 2014). However, the existing body of evidence is primarily derived from studies conducted in Western populations. Given the potential for significant differences in genetic predisposition, clinical characteristics, epidemiological patterns, and standard medication practices, the generalizability of these findings to Chinese patients remains uncertain. A critical evidence gap exists regarding the specific risk profile for hypoglycemia in Chinese inpatient settings.

This study was designed to specifically assess the incidence of hypoglycemia and investigate factors associated with hypoglycemia in a cohort of hospitalized Chinese patients with diabetes. Its findings hold the prospect of paving the way for evidence-based, population-specific interventions aimed at mitigating hypoglycemia risk and improving standards of inpatient diabetes management.

## 2 Methods

### 2.1 Patients and data collection

Our study retrospectively analyzed the electronic medical records of diabetes patients admitted to Nanfang Hospital of Southern Medical University from January 2021 to December 2022. The inclusion criteria were as follows: (1) aged ≥18 years and (2) diagnosed with T1DM or T2DM. The exclusion criteria were as follows: (1) patients diagnosed with hypoglycemia upon admission; (2) patients without blood glucose monitoring results during hospitalization.

### 2.2 Study population and data extraction

We defined Level 1 hypoglycemia as a blood glucose (capillary and venous plasma glucose) concentration of ≤3.9 mmol/L (70 mg/dL). In line with clinical guidelines and to further characterize event severity, we also identified clinically significant (Level 2) hypoglycemia as a plasma glucose concentration of <3.0 mmol/L (54 mg/dL). In our hospital, capillary blood glucose monitoring is performed according to a standardized protocol. For all hospitalized patients with diabetes, the routine order for capillary glucose testing is eight times daily (before and after each meal, at bedtime, and as needed), regardless of the patient’s medication regimen or clinical stability. This standardized schedule ensures uniform monitoring frequency across the study population.

In order to ensure temporal precedence and establish a potential predictive relationship, the following criteria were applied to data extraction.

1. Baseline demographic characteristics and comorbidities for all patients were collected from admission records, as these represent pre-existing conditions that were present prior to and unaffected by hospitalization.
2. For patients who experienced hypoglycemia, all laboratory parameters and medication use were required to be documented in the electronic medical record (EMR) before the onset of their first recorded hypoglycemic event.
3. For patients with multiple hypoglycemic episodes, predictors were exclusively drawn from data preceding the initial event.
4. For patients without any hypoglycemic event, laboratory and medication data were extracted from their entire hospitalization period.

This temporal restriction was implemented to establish a clear temporal relationship between exposures and outcome, thereby minimizing the risk of reverse causality (i.e., medication changes made in response to hypoglycemia).

The selection of variables for analysis was informed by previous literature on hypoglycemia risk factors. We collected the following data, categorized into three groups (Missing data for each variable are reported in Supplementary Table S1. Variables with a missing rate exceeding 30% were excluded from the analysis):

Demographics and behavioral characteristics: age, sex, diabetes type, BMI, smoking history, and alcohol use.

Comorbidities: including heart failure, renal insufficiency, and hepatic dysfunction, malnutrition, dementia, and postoperative status.

Treatment and Laboratory Parameters: use of antidiabetic medications (e.g., insulin, sulfonylureas, metformin); and laboratory values such as glycosylated hemoglobin (HbA1c), renal function tests (e.g., creatinine), liver function tests, and routine blood parameters. We included commonly available inpatient tests like average glucose, HbA1c, creatinine (CR), C-reactive protein (CRP), hemoglobin (HGB) and proteinuria (PRO) to identify readily obtainable laboratory predictors of hypoglycemia.

### 2.3 Statistical analyses

Data analysis was conducted via the statistical software SPSS version 27.0. The normally distributed measurement data are presented as the means and standard deviations (SDs), and the two patient groups were compared via a nonparametric Mann‒Whitney U test. The enumeration data are expressed as n (%) and were analyzed with a χ2 test, and multivariate analysis was carried out via binary logistic regression, with a p‐value less than 0.05 considered statistically significant. The independent variables were those that were statistically significant in the univariate analysis (p < 0.05) and those deemed significant on the basis of clinical expertise, whereas the dependent variable was the classification of hypoglycemia (treated as a binary variable). The threshold for the inclusion of variables was a p value of 0.05. Factors associated with hypoglycemia were identified based on odds ratios (ORs), and the results are reported with the ORs and 95% confidence intervals (CIs).

## 3 Results

### 3.1 General data comparison

**FIGURE 1:**
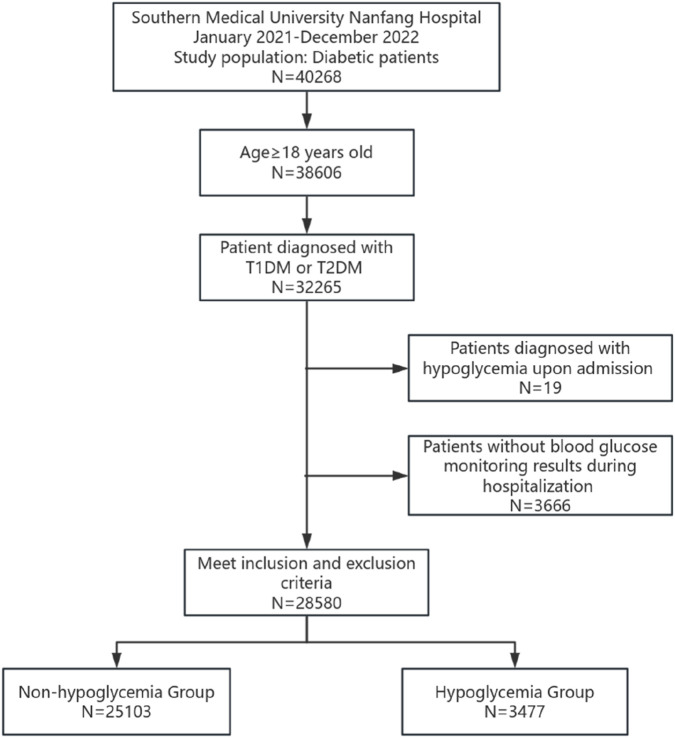
The workflow of patient enrollment. After the inclusion and exclusion criteria were applied, a total of 28,580 patients were enrolled in the study, including 3,477 in the hypoglycemia group and 25,103 in the nonhypoglycemia group. N, number of individuals; T1DM, type 1 diabetes mellitus; T2DM, type 2 diabetes mellitus.

A total of 40,268 patients with diabetes were hospitalized during the study period. Of these, 32,265 had a confirmed diagnosis of T1DM or T2DM. After applying the inclusion and exclusion criteria, 28,580 patients were included in the final study cohort. Among the enrolled patients, 3,477 (12.2%) experienced at least one hypoglycemic event (≤3.9 mmol/L) during their hospitalization (Figure 1). Of these, 538 patients (1.8%) experienced at least one episode of Level 2 hypoglycemia (<3.0 mmol/L). The baseline characteristics of the cohort are presented in Table 1. The study population had a mean age of 60.47 ± 11.76 years and a mean BMI of 24.05 ± 3.73 kg/m^2^. Females constituted 35.4% of the cohort, and the vast majority (99.0%) were diagnosed with T2DM (Table 1).

**TABLE 1:** Baseline characteristics of the study population.

​	Total(N = 28580)
Characteristic	Mean or N (%)
^a^Age	60.47 ± 11.76
^b^Sex(female), n(%)	10120(35.4)
^b^Type of diabetes, n(%)	​
T1DM	293(1.0)
T2DM	28287(99.0)
^b^ Smoking(0), n(%)	15624(54.7)
^b^Alcohol(0), n(%)	18172(63.6)
^a^BMI, kg/m^2^	24.05 ± 3.73
^b^Hypoglycemia, n(%)	3477(12.2%)
^b^Level 2 Hypoglycemia, n(%)	538(1.8%)

The data are presented as numbers (%), means ± SDs.

N, number of individuals; T1DM, type 1 diabetes mellitus; T2DM, type 2 diabetes mellitus; BMI, body mass index.

### 3.2 Risk factor analysis

Results of the univariate analysis are presented in Table 2. Several factors demonstrated significant associations with hypoglycemic events. Regarding demographics and behavioral characteristics, younger age, a diagnosis of T1DM (versus T2DM) (p < 0.001), a history of alcohol consumption, and a lower BMI were associated with a higher risk of hypoglycemia. In laboratory findings, the NON-HG group had higher average glucose levels (p < 0.001). Higher levels of CRP (p < 0.001) and CR (p < 0.001) were associated with increased risk, whereas higher levels of HGB (p < 0.001) and the absence of PRO (p < 0.001) were associated with decreased risk. Concerning medications, the use of SGLT-2 inhibitors (SGLT-2i), DPP-4 inhibitors (DPP-4i), GLP-1 receptor agonists (GLP-1ra), AGIs, or metformin was associated with a lower risk of hypoglycemia (both p < 0.001). Conversely, the use of glinides or insulin was associated with a substantially higher risk (both p < 0.001). The analysis did not identify any significant associations between the studied comorbidities and hypoglycemia, which may be attributable to their overall low prevalence in the cohort or limited statistical power for these specific conditions.

**TABLE 2:** Univariable analysis of demographic and clinical factors associated with hypoglycemic risk.

Characteristic	NON-HG (25103)	HG (3477)	P
^a^Age	60.55 ± 11.68	59.88 ± 12.30	0.035*
^b^Sex, female, n(%)	8847(35.2)	1273(36.6)	0.114
^b^Diabetes(T2DM), n(%)	24940(99.4)	3347(96.3)	<0.001***
^b^Smoking(0), n(%)	13648(54.4)	1976(56.8)	0.294
^b^Alcohol(0), n(%)	15816(63.0)	2356(67.8)	<0.001***
^a^BMI, kg/m^2^	24.18 ± 3.72	23.08 ± 3.66	<0.001***
Laboratory examinations			
^a^Average glucose, mmol/L	9.03 ± 2.87	8.39 ± 2.99	<0.001***
^a^HbA1c, %	7.74 ± 1.94	7.78 ± 2.11	0.571
^a^HGB, g/L	121.03 ± 23.91	110.89 ± 25.36	<0.001***
^a^CRP, mg/L	19.81 ± 33.35	27.51 ± 43.31	<0.001***
^a^UA, μmol/L	340.82 ± 111.29	342.23 ± 120.20	0.781
^b^PRO(−), (%)	15882(63.3)	1665(47.9)	<0.001***
^a^CR, μmol/L	121.83 ± 156.42	190.80 ± 239.50	<0.001***
Medicine			
^b^SU (%)	2234(8.9)	334(9.6)	0.172
^b^SGLT-2i (%)	6063(24.2)	502(14.4)	<0.001***
^b^Glinide (%)	718(2.9)	213(6.1)	<0.001***
^b^DPP-4i (%)	4557(18.2)	462(13.3)	<0.001***
^b^GLP-1ra (%)	882(3.5)	66(1.90)	<0.001***
^b^AGI (%)	5521(22.0)	545(15.7)	<0.001***
^b^TZDs (%)	454(1.8)	71(2.0)	0.337
^b^Insulin (%)	11783(46.9)	2325(66.9)	<0.001***
^b^Metformin (%)	8984(35.8)	815(23.4)	<0.001***
Comorbidity			
^b^Heart failure (%)	1056(4.2)	163(4.7)	0.188
^b^Renal insufficiency (%)	2200(8.8)	323(9.3)	0.306
^b^Hepatic insufficiency (%)	256(1.0)	25(0.7)	0.092
^b^Malnutrition (%)	95(0.4)	7(0.2)	0.101
^b^Dementia (%)	142(0.6)	17(0.5)	0.569
^b^Postoperative (%)	4387(17.5)	583(16.8)	0.301

The data are presented as the means ± standard deviations or percentages.

Abbreviations: HG, hypoglycemia; T1DM, Type 1 diabetes mellitus; T2DM, Type 2 diabetes mellitus; BMI, body mass index; HbA1c, Glycosylated hemoglobin; HGB, hemoglobin; CRP, C-reactive protein; UA, uric acid; PRO, proteinuria; CR, creatinine; SU, sulfonylurea; SGLT-2i, Sodium-dependent glucose transporter 2 inhibitors; DPP-4i, Dipeptidyl peptidase-4 inhibitors; GLP-1ra, Glucagon-like peptide-1 receptor agonists; AGI, Alpha-glucosidase inhibitor; TZDs, Thiazolidinediones.

^a^ p‐values from the nonparametric Mann–Whitney U test.

^b^ p‐value from the Pearson c2 test.

*p < 0.05; **p < 0.01; ***p < 0.001.

A multivariable logistic regression analysis was performed in Table 3, incorporating the significant variables from the univariate analysis. Following adjustment for other covariates, a diagnosis of T1DM was found to be independently associated with a significantly higher risk of hypoglycemia compared to T2DM, with an odds ratio (OR) of 4.11 (p < 0.001). Furthermore, increased age (p = 0.004) and higher BMI (p < 0.001) were identified as protective factors, with each unit increase associated with a reduction in hypoglycemia risk. Additionally, a history of alcohol consumption was also protective (p = 0.002). In laboratory parameters, higher average glucose level and HGB levels were significantly associated with a decreased risk of hypoglycemia (p < 0.001). Conversely, elevated CR levels and the presence of PRO were both independent risk factors for hypoglycemia (both p < 0.001). CRP was not significantly associated with hypoglycemia in the multivariable analysis (p = 0.229). Regarding glucose-lowering medications used during hospitalization, we found that the use of glinides and insulin was associated with a significantly elevated risk of hypoglycemia, approximately doubling the risk (both p < 0.001). In contrast, the use of other classes of antidiabetic drugs was associated with lower odds of hypoglycemia or had no association.

**TABLE 3:** Multivariate binary logistic regression analysis for hypoglycemia in hospitalized patients with diabetes mellitus.

Variables	B	SE	Wald	p‐Value	OR	95% CI
Age	−0.007	0.002	8.248	0.004 **	0.993	0.989–0.998
Diabetes(T2DM)	1.413	0.178	62.968	<0.001 ***	4.11	2.899–5.827
Alcohol	−0.181	0.058	9.845	0.002 **	0.835	0.746–0.934
BMI	−0.087	0.008	126.449	<0.001 ***	0.917	0.903–0.931
Average Glucose	−0.128	0.011	147.346	<0.001 ***	0.88	0.862–0.898
HGB	−0.004	0.001	11.624	<0.001 ***	0.996	0.993–0.998
CRP	0.001	0.001	1.449	0.229	1.001	0.999–1.003
PRO (−)	0.346	0.064	29.023	<0.001 ***	1.414	1.246–1.604
CR	0.001	0	40.412	<0.001 ***	1.001	1.001–1.001
SGLT-2i (−)	−0.188	0.071	7.073	0.008 **	0.829	0.722–0.952
Glinide (−)	0.718	0.113	40.604	<0.001 ***	2.051	1.644–2.558
DPP-4i (−)	−0.348	0.074	22.121	<0.001 ***	0.706	0.61–0.816
GLP-1ra (−)	−0.064	0.158	0.162	0.687	0.938	0.688–1.279
AGI (−)	−0.195	0.068	8.235	0.004 **	0.823	0.72–0.94
Insulin (−)	0.947	0.059	255.652	<0.001 ***	2.578	2.295–2.895
Metformin (−)	−0.119	0.062	3.693	0.055	0.888	0.787–1.002

Abbreviations: T2DM, type 2 diabetes mellitus; BMI, body mass index; HbA1c, glycosylated hemoglobin; HGB, hemoglobin; CRP, C-reactive protein; UA, uric acid; PRO, proteinuria; CR, creatinine; SGLT-2i, sodium-dependent glucose transporter 2 inhibitors; DPP-4i, dipeptidyl peptidase-4 inhibitors; GLP-1ra, glucagon-like peptide-1 receptor agonists; AGI, alpha-glucosidase inhibitor.

*p < 0.05; **p < 0.01; ***p < 0.001.

### 3.3 Temporal characteristics of hypoglycemia

Among the 3,477 patients with hypoglycemia, a total of 6,101 hypoglycemic events were documented during hospitalization (4,547 detected by capillary glucose testing and 1,554 by venous plasma glucose testing). The temporal distribution of these events was analyzed separately for T1DM and T2DM.

**FIGURE 2:**
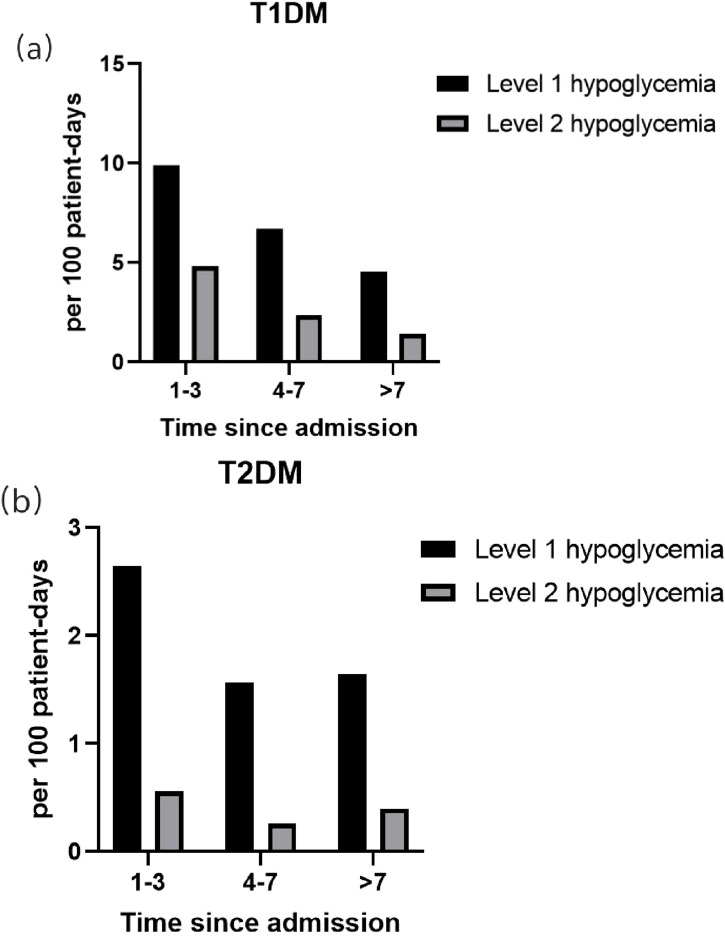
Incidence density and distribution of hypoglycemic events during hospitalization. **(a)** Patients with type 1 diabetes (T1DM); **(b)** patients with type 2 diabetes (T2DM). The bar chart presents the incidence density (events per 100 patient-days, y-axis). Data are shown across three hospitalization periods: 1–3 days, 4–7 days, and >7 days.

As shown in Figure 2, which presents both the percentage and the incidence density (events per 100 patient-days) of hypoglycemia, distinct patterns emerged between the two types. For patients with T1DM, hypoglycemia occurred predominantly within the first 3 days of admission, with an incidence density of 9.89 events/100 patient-days for Level 1 hypoglycemia. The incidence density declined steadily thereafter during days 4–7 and beyond 7 days (Level 1: 6.68 vs. 4.56 events/100 patient-days). For patients with T2DM, the pattern differed, characterized by a lower and more stable incidence density throughout the hospitalization. Although the highest proportion of events occurred in the first 3 days (29.0%), the corresponding incidence density was substantially lower (Level 1: 2.64 events/100 patient-days) and remained low in subsequent periods (Level 1: 1.57 events/100 patient-days on days 4–7 and 1.65 events/100 patient-days after 7 days).

**FIGURE 3:**
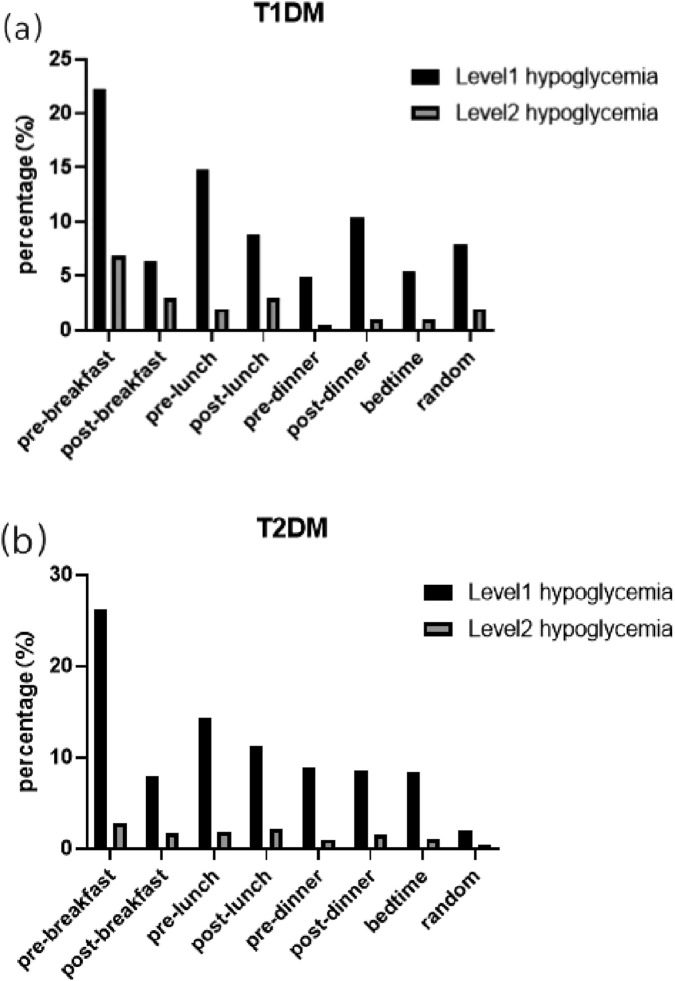
Time characteristics of hypoglycemia in hospitalized patients with diabetes. **(a)** Type 1 diabetes (T1DM); **(b)** type 2 diabetes (T2DM). This bar chart depicts the percentage of all hypoglycemic events occurring at specific times of the day. Events are categorized by severity: Level 1 (≤3.9 mmol/L, black bars) and Level 2 (≤3.0 mmol/L, gray bars). The time points include pre- and post-meal periods as well as random measurements.

Regarding the time of day (Figure 3), the patterns for T1DM and T2DM were similar. For both groups, the pre-breakfast period was the most common time for hypoglycemia (22.17% and 26.27% of events for T1DM and T2DM, respectively), followed by the pre-lunch period (14.78% and 14.36%, respectively).

### 3.4 Subgroup analyses

To further characterize the study population, we conducted two additional subgroup analyses. Among the 3,477 patients who experienced hypoglycemia, 1,222 (35.2%) had two or more documented episodes during their hospitalization. A comparison of key characteristics between patients with a single episode (n = 2,255) and those with multiple episodes is presented in Table 4. Patients with recurrent hypoglycemia were significantly younger (59.51 ± 12.08 vs. 60.55 ± 12.68 years, p = 0.018), had a lower proportion of T2DM (95.0% vs. 96.9%, p < 0.004), a lower BMI (22.71 ± 3.71 vs. 23.26 ± 3.62 kg/m^2^, p < 0.001), and a higher rate of insulin use (76.4% vs. 61.7%, p < 0.001). Sex distribution did not differ significantly between the groups.

**TABLE 4:** Characteristics of patients with single versus multiple hypoglycemic episodes.

Variables	Single episode (2255)	Multiple episodes (1222)	P
^a^Age	60.55 ± 12.68	59.51 ± 12.08	0.018*
^b^Sex, female, n(%)	835(37.0)	438(35.8)	0.488
^b^Diabetes(T2DM), n(%)	2186(96.9)	1161(95.0)	<0.004 **
^a^BMI,kg/m2	23.26 ± 3.62	22.71 ± 3.71	<0.001 ***
^b^Insulin (%)	1391(61.7)	934(76.4)	<0.001 ***

Abbreviations: T2DM, type 2 diabetes mellitus; BMI, body mass index.

^a^ p‐values from the nonparametric Mann–Whitney U test.

^b^ p‐value from the Pearson c2 test.

*p < 0.05; **p < 0.01; ***p < 0.001.

**FIGURE 4:**
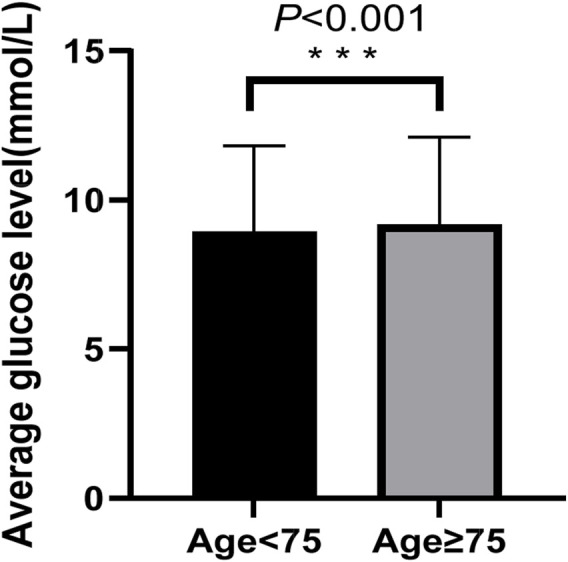
Average glucose levels by age group. The comparison was performed using the Independent Samples T-test. *** denotes p < 0.001.

Given that older age was identified as a protective factor against hypoglycemia in the primary analysis, we sought to explore whether differences in glycemic management might underlie this association. Therefore, we compared the mean blood glucose levels during hospitalization between patients aged ≥75 years and those <75 years. Older patients (≥75 years) had a significantly higher average glucose level than younger patients (9.17 ± 2.93 mmol/L vs. 8.93 ± 2.89 mmol/L, p < 0.001; Figure 4).

## 4 Discussion

Hypoglycemia remains a major barrier to stringent glycemic control. Our 2 year retrospective analysis found an in-hospital hypoglycemia incidence of 12.2% (3,477/28,580), a rate consistent with reports from Western countries (Pratiwi et al., 2020; Gómez-Huelgas et al., 2015).

Our study identified several key factors associated with hypoglycemia. Most notably, a diagnosis of T1DM was the strongest predictor, associated with a 4.11-fold higher risk of hypoglycemia compared to T2DM. This is likely due to the absolute insulin deficiency in T1DM, necessitating lifelong insulin therapy, which inherently increases hypoglycemia risk (Carey et al., 2013; Chen et al., 2023). Older age and higher BMI were identified as protective factors. While the physiological relationship between age and hypoglycemia remains debated, our finding that older age is protective aligns with analyses from large studies such as the multinational HAT study (Khunti et al., 2016). Our subgroup analysis offers a plausible explanation for this association: patients aged ≥75 years had significantly higher average glucose levels during hospitalization compared to younger patients. This strongly suggests that the observed protective effect is not a direct physiological consequence of aging but likely reflects prevalent, less stringent glycemic management practices in older inpatients, consistent with guideline recommendations for individualized targets. Similarly, the protective effect of higher BMI may be explained by greater insulin resistance in patients with obesity, which can buffer against glucose-lowering therapies (Yun et al., 2019).

Regarding glucose-lowering medications, the use of glinides and insulin was associated with significantly higher odds, a well-established finding in previous research (Kostev et al., 2014; Silbert et al., 2018). Conversely, the use of other drug classes, including SGLT-2 inhibitors, DPP-4 inhibitors, AGIs, and metformin, was associated with lower odds of hypoglycemia. The safety profile of these agents, particularly SGLT-2i and metformin, is consistent with their position as first-line recommendations in current T2DM management guidelines (Ligthelm et al., 2012; Kim et al., 2025). These findings may be susceptible to the healthy adherer effect, where patients who receive and adhere to newer antidiabetic agents may have healthier behaviors and better overall disease management (Prada-Ramallal et al., 2019; Shrank et al., 2011). We found no significant association between the assessed comorbidities and hypoglycemia, possibly due to their low prevalence in our study cohort.

We also incorporated laboratory parameters and found that worse renal function, indicated by elevated CR levels or the presence of PRO, was a significant independent risk factor. Conversely, higher hemoglobin levels were protective. Compared to a categorical diagnosis of “renal insufficiency,” actual laboratory values like CR and PRO provide a more sensitive and dynamic reflection of current renal status (Horton et al., 2022; Ooi et al., 2023). Therefore, we posit that these objective laboratory data could be highly valuable for constructing future risk prediction models for in-hospital hypoglycemia.

Furthermore, our analysis of the temporal distribution of hypoglycemia, normalized for length of stay, revealed distinct patterns. Hypoglycemia in T1DM patients was heavily concentrated within the first 3 days of admission, with the highest incidence density (Level 1: 9.89 events/100 patient-days), declining sharply thereafter (days 4–7 vs. after 7 days: Level 1: 6.68 vs. 4.56 events/100 patient-days). In contrast, the pattern for patients with T2DM differed, characterized by a lower and more stable incidence density throughout the hospitalization. Although the incidence density was also highest in the first 3 days (Level 1: 2.64 events/100 patient-days), it remained at similarly low levels in the subsequent periods (Level 1: 1.57 events/100 patient-days on days 4–7 and 1.65 events/100 patient-days after 7 days). This indicates an acute, early risk for T1DM, whereas for T2DM, the risk persists at a lower absolute level. Regarding the time of day, both groups exhibit similar patterns, with the period pre-breakfast being the highest risk time. These patterns may be attributed to circadian rhythm-related counterregulatory hormone deficiencies (e.g., nocturnal glucagon decline) and prolonged intermeal intervals during hospitalization (Li et al., 2023; Bailon et al., 2009). These distinct temporal patterns underscore the need for tailored monitoring strategies—vigilant early surveillance for T1DM and sustained awareness throughout the stay for T2DM, with particular attention to the pre-breakfast period for all.

Capillary blood glucose was measured eight times daily for all patients according to routine protocol, regardless of their treatment regimen or clinical stability. This uniform monitoring frequency minimized the risk of detection bias for capillary glucose measurements. Although venous plasma glucose tests were ordered at physicians’ discretion and may have been obtained more frequently in unstable patients, the majority of hypoglycemic events (4,547/6,101, 74.5%) were captured by the standardized capillary tests. Therefore, any potential detection bias related to differential venous sampling is unlikely to have a substantial impact on our findings.

Additionally, we characterized patients who experienced multiple hypoglycemic events, a high-risk subgroup constituting 35.1% of all patients with hypoglycemia. These individuals were more likely to be younger, have T1DM, have a lower BMI, and be treated with insulin, reinforcing the factors identified in our primary analysis and confirming a distinct, vulnerable population requiring intensive management.

The limitations of this study are as follows. 1) First, this was a retrospective study. Although we focused on medication exposure prior to hypoglycemic events to minimize reverse causality, medications were analyzed as binary variables without accounting for dosage, duration, or timing of initiation. Additionally, we could not obtain patients’ pre-admission medication information, which may be related to the high incidence of hypoglycemia within the first 3 days after admission. 2) Hypoglycemia is associated with changes in exercise intensity, patient dietary intake, and prolonged fasting, but we cannot obtain information about the patient’s condition at the time (Carey et al., 2013; Bae et al., 2017). 3) In a single hospital, the study included patients with some heterogeneity in disease severity, age distribution, and individualized treatment plans. Thus, future studies should incorporate more hospitals. 4)Although we reported the incidence of clinically significant (Level 2) hypoglycemia, the number of such events limited our ability to perform a separate, statistically robust multivariable analysis to identify risk factors unique to severe hypoglycemia. Larger cohort studies are warranted to address this question.

Overall, this study confirms a clinically significant incidence of hypoglycemia in the inpatient diabetes population. The identification of specific risk profiles and temporal trends directly informs the development of proactive, evidence-based management protocols to prevent hypoglycemic events and improve the quality of care.

## Data Availability

The data analyzed in this study is subject to the following licenses/restrictions: The datasets used and analyzed during the current study are not publicly available due to hospital policy and patient privacy regulations but are available from the corresponding author on reasonable request. Requests to access these datasets should be directed to PZ, zpm321@126.com.
