# Supplementary material for: Risk factors for hypoglycemia in hospitalized patients with diabetes: a retrospective analysis of electronic medical record data
Source: Front Pharmacol. 2026 Mar 26;17:1741870. doi: 10.3389/fphar.2026.1741870 (PMC13061714; doi:10.3389/fphar.2026.1741870)
Supplement: Supplementary file 1 [file Table1.docx]

Table S1. Missing data for each variable in the study cohort (N=28,580)

| Valuable name | Missing rate |
| --- | --- |
| Sex | 0 (0%) |
| Diabetes | 0 (0%) |
| Smoking | 2116 (7.4%) |
| Alcohol | 2412(8.4%) |
| BMI | 3037(10.6%) |
| Average glucose | 0(0%) |
| HbA1c | 8544(29.9%) |
| HGB | 749(2.6%) |
| CRP | 6352(22.2%) |
| UA | 1309(4.6%) |
| PRO | 4825(16.9%) |
| CR | 1109(3.9%) |
| Cystatin C | 13694(47.9%) |
| 24h PRO | 21971(76.9%) |
| SU | 0 (0%) |
| SGLT-2i | 0 (0%) |
| Glinide | 0 (0%) |
| DPP-4i | 0 (0%) |
| GLP-1ra | 0 (0%) |
| AGI | 0 (0%) |
| TZDs | 0 (0%) |
| Insulin | 0 (0%) |
| Metformin | 0 (0%) |
| Heart failure | 0 (0%) |
| Renal insufficiency | 0 (0%) |
| Hepatic insufficiency | 0 (0%) |
| Malnutrition | 0 (0%) |
| Dementia | 0 (0%) |
| Postoperative | 0 (0%) |

Abbreviations: BMI: Body mass index; HbA1c: Glycosylated hemoglobin; HGB: Hemoglobin; CRP: C-reactive protein; UA: Uric acid; PRO: Proteinuria; CR: Creatinine; SU: Sulfonylurea; SGLT-2i: Sodium-dependent glucose transporter 2 inhibitors; DPP-4i: Dipeptidyl peptidase-4 inhibitors; GLP-1ra: Glucagon-like peptide-1 receptor agonists; AGI: Alpha-glucosidase inhibitor; TZDs: Thiazolidinediones;
